# Pathways to ensure universal and affordable access to hepatitis C treatment

**DOI:** 10.1186/s12916-018-1162-z

**Published:** 2018-10-09

**Authors:** Caitlin H. Douglass, Alisa Pedrana, Jeffrey V. Lazarus, Ellen F. M. ‘t Hoen, Radi Hammad, Ricardo Baptista Leite, Andrew Hill, Margaret Hellard

**Affiliations:** 1 Burnet Institute, Melbourne, Australia; 2 Barcelona Institute for Global Health (ISGlobal), Hospital Clínic, University of Barcelona, Barcelona, Spain; 3 CHIP, Rigshospitalet, University of Copenhagen, Copenhagen, Denmark; 4 Global Health Unit, University Medical Centre, Groningen, The Netherlands; 5 Medicines Law and Policy, Amsterdam, The Netherlands; 6 National Hepatology and Tropical Medicine Research Institute, Cairo, Egypt; 7 Universidade Católica Portuguesa, Lisbon, Portugal; 8 Faculty of Health, Medicine and Life Sciences, Maastricht University, Maastricht, The Netherlands; 9 Department of Molecular and Clinical Pharmacology, Liverpool University, Liverpool, UK; 10 Department of Infectious Diseases, The Alfred Hospital, Melbourne, Australia; 11 Department of Epidemiology and Preventive Medicine, Monash University, Melbourne, Australia; 12 Doherty Institute, University of Melbourne, Melbourne, Australia

**Keywords:** Healthcare financing, Hepatitis C, Treatment

## Abstract

Direct-acting antivirals (DAAs) have dramatically changed the landscape of hepatitis C treatment and prevention. The World Health Organization has called for the elimination of hepatitis C as a public health threat by 2030. However, the discrepancy in DAA prices across low-, middle- and high-income countries is considerable, ranging from less than US$ 100 to approximately US$ 40,000 per course, thus representing a major barrier for the scale-up of treatment and elimination. This article describes DAA pricing and pathways to accessing affordable treatment, providing case studies from Australia, Egypt and Portugal. Pathways to accessing DAAs include developing comprehensive viral hepatitis plans to facilitate price negotiations, voluntary and compulsory licenses, patent opposition, joint procurement, and personal importation schemes. While multiple factors influence the price of DAAs, a key driver is a country’s capacity and willingness to negotiate with pharmaceutical companies. If negotiations do not lead to a reasonable price, governments have the option to utilise flexibilities outlined in the Agreement on Trade-Related Aspects of Intellectual Property Rights. Affordable access to DAAs is underpinned by collaboration between government, civil society, global organisations and pharmaceutical companies to ensure that all patients can access treatment. Promoting these pathways is critical for influencing policy, improving access to affordable DAAs and achieving hepatitis C elimination.

## Background

An estimated 67 million people live with chronic hepatitis C infection worldwide [1]. Chronic hepatitis C causes cirrhosis, liver cancer and approximately 399,000 deaths annually [2]. Globally, people who inject drugs (PWID) have the highest hepatitis C prevalence (42–62%) [3]. In 2016, the World Health Organization (WHO) set targets for eliminating hepatitis C as a public health threat, calling for an 80% reduction in incidence and a 65% reduction in related deaths by 2030 [4]. WHO estimates that approximately 1.5 million new hepatitis C infections occur annually [1], yet, in 2017, only 1.6 million patients were treated for hepatitis C and fewer than 1.5 million are expected to be treated in 2018 [1]. If current trajectories of new infections and treatment uptake continue, hepatitis C will certainly not be eliminated by 2030 and only 12 countries are likely to achieve elimination [1]. Therefore, a multi-pronged approach that includes the scale-up of affordable treatment is essential to achieve elimination [5–7].

The advent of direct-acting antivirals (DAAs) in 2013 led to optimism that hepatitis C elimination is achievable [8]. DAAs have a cure rate of over 95%, treatment duration of only 8–12 weeks, and fewer and less severe side-effects than their predecessors [2]. However, in many countries, DAA prices discourage treatment, impeding progress towards elimination [9, 10], and whilst DAA prices have declined, they vary considerably – from less than US$ 100 per treatment course to approximately US$ 40,000 [9, 11, 12]. This paper describes current pricing of DAAs and highlights the pathways and mechanisms for governments and civil society to access affordable treatment.

## Drug development

The advancing of drugs through research and development (R&D) is a long-term process, with an only 12% probability of a drug gaining marketing approval [13]. Estimates of the average R&D cost vary from US$ 161 million [14] to US$ 2.6 billion per drug, including approved and unsuccessful compounds and opportunity costs [13]. Additional costs post-approval include surveillance, manufacturing, distribution and marketing.

## Marketing authorisation

Following clinical trials, drug companies must obtain marketing authorisation to sell their products (e.g. through the Food and Drug Administration (FDA) or European Medicines Agency). Over 2011–2015, the FDA and European Medicines Agency median approval times for new therapeutic agents were 306 and 383 days, respectively [15]. Accelerated review processes apply for important medicines that treat serious conditions and fill an unmet need or demonstrate substantial improvements over existing treatments [16]. For example, the FDA granted sofosbuvir, a highly effective DAA manufactured by Gilead Sciences, a priority review [17], and it was approved for the treatment of chronic hepatitis C within 242 days of application [18].

## DAA pricing

Upon marketing approval, decisions on pricing and reimbursement are required. These decisions vary according to whether governments negotiate discounts or whether pharmaceutical companies set prices [19]. In 2013, the price of sofosbuvir ranged from US$ 900 in Egypt to US$ 95,000 in the US [11, 12, 20]. Since then, other DAAs have been released and generic DAAs have begun to generate competition, leading to discounted prices [9]. Additionally, manufacturing costs have also fallen, with the costs of a 12-week course of sofosbuvir estimated at US$ 47 [21].

DAAs contribute significantly to pharmaceutical company profits. In 2017, Gilead reported US$ 26.1 billion in sales, including US$ 9.1 billion from DAAs [22]. In 2017, Bristol-Myers Squibb’s product revenue totalled US$ 20.8 billion, including US$ 406 million from hepatitis C products [23]. Whilst the exact costs of DAA R&D, manufacturing and distribution are unknown, sales data suggest that pharmaceutical companies make significant profits from DAAs, particularly from high-income countries.

The initial price of DAAs was particularly daunting as it required governments to dedicate large proportions of their health budget to hepatitis C treatment [24]. However, despite DAA manufacturing costs falling, prices still vary, remaining stubbornly high in certain countries [11]. A number of high-income countries, including Australia, Finland, France, Iceland, Italy, Norway, Portugal, Scotland, Spain and Sweden, have negotiated price agreements that allow most patients to access DAAs, enabling a broad public health response rather than a disease-staged approach [12]. In 2015, Georgia, a middle-income country, established the world’s first hepatitis C elimination demonstration project, with Gilead Sciences providing free treatment for all patients [25]. Georgia’s elimination plan derived from strong political commitment, public support and health system capacity [26].

Other countries (e.g. US [10], Denmark and Poland [12]) are paying higher prices for DAAs, necessitating treatment restrictions based on fibrosis stage and substance use [27, 28]. A review showed that, of 35 European countries and jurisdictions, 46% restricted treatment to patients with fibrosis at stage F2 or higher and 17% required abstinence from illicit substances [28]. Importantly, these restrictions were not based on treatment effectiveness, with DAAs achieving > 95% cure rates in patients without cirrhosis or with compensated cirrhosis, PWID and those who consume alcohol [29–32] and 78–87% cure rates for patients with compensated cirrhosis [33]. Additionally, the key to preventing new infections is stopping viral transmission. Mathematical modelling demonstrates that PWID with hepatitis C must be treated to reduce incidence and reach WHO elimination targets [34, 35]. Hence, treatment restrictions for PWID and other priority groups must be lifted.

## Pathways to accessing DAAs

**Fig. 1:**
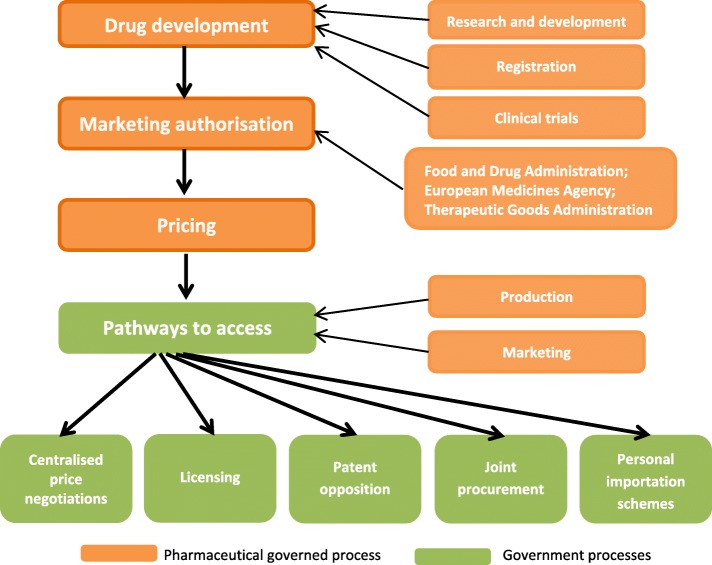
Pathways to accessing direct-acting antivirals

Whilst DAA price reductions in some countries facilitate greater treatment access, prices need to be affordable worldwide. A government’s capacity to negotiate with pharmaceutical companies is a key driver of drug prices. Other pathways to accessing affordable DAAs include voluntary and compulsory licensing, patent opposition, personal importation schemes, and joint procurement (Fig. 1).

## Centralised price negotiations

In the US, some patients are eligible for DAAs through Medicare (federal health insurance for people who are older or disabled) and Medicaid (government-subsidised healthcare programmes for people with low income) [36]. While Medicare cannot negotiate drug prices directly [9], Medicaid has previously obtained manufacturer rebates for HIV drugs above the required Medicaid price discounts for all drugs. The Veterans Health Administration can negotiate deals for veterans and private insurance companies can negotiate for clients [37]. However, strict confidentiality agreements between payers and manufacturers prevent price transparency [36]. Consequently, US patients pay substantially more for DAAs than citizens of other high-income countries, thus reducing treatment access [9]. US states and insurance companies vary considerably regarding DAA restrictions and reimbursement based on fibrosis stage, substance use, co-infection with HIV and prescriber type [38]. However, several states have relaxed their DAA criteria, allowing increased treatment utilisation [39].

Despite restrictions on drug price negotiation in the US, legislation (under 28 U.S.C. §1498) permits the government to purchase generic medications at less than 1% of their branded list price plus a reasonable royalty [37]. During the 2001 anthrax outbreak, the US Government threatened to use this legal provision, leading to swift negotiations with the manufacturer for reduced medication costs [37]. This mechanism could be used to reduce DAA prices.

In other countries, governments can negotiate a reasonable price for pharmaceuticals with patent holders [40]. Australia and Portugal have negotiated volume-based agreements with pharmaceutical companies (case studies 1 and 2), and Italy and Spain have negotiated DAA course prices of less than € 8000. It is apparent that pharmaceutical companies are willing to negotiate reasonable prices when governments present plans that ensure a greater number of patients undertake treatment.

## Intellectual property law

In 1994, the World Trade Organization (WTO) established the Agreement on Trade-Related Aspects of Intellectual Property Rights (TRIPS) [40]. The TRIPS Agreement sets global minimum requirements for creating and protecting intellectual property. WTO members, excluding those regarded as least developed countries (LDCs) (low-income countries at high risk of economic and environmental shocks), must provide patent protection for inventions for at least 20 years [41]. Although patents provide an incentive to innovate, they can also create monopolies, reduce competition and increase prices [42]. Patent holders can prevent generic versions of patented medicines from being produced or made available. However, LDCs are not obliged to provide or enforce medicine patents or data protection until at least 2033, allowing the legal production and marketing of generic medicines in these countries [43].

**Fig. 2:**
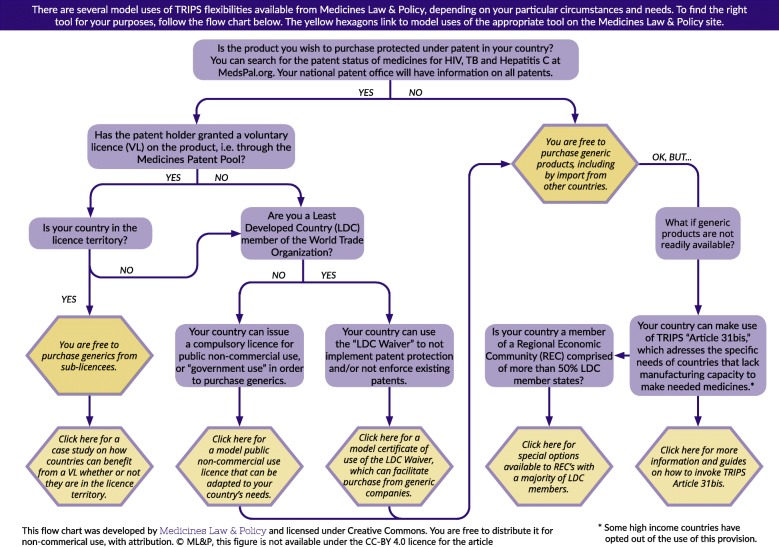
TRIPS flexibilities retrieved from Medicines Law & Policy

The basic compound patent for sofosbuvir expires in a number of patent-granting countries in 2025 [44]. Upon patent expiry, generics can be freely produced and disseminated, creating competition and reducing costs for governments and consumers [45, 46]. For example, generic competition for HIV antiretroviral therapy (ART) contributed to a 99% price reduction [47]. The TRIPS Agreement also enables governments to prevent a patent’s monopoly effects and access generics prior to patent expiry (Fig. 2) [41]. In 2001, the WTO adopted the Doha Declaration on TRIPS and Public Health, clarifying that TRIPS should not hamper governments’ ability to protect public health and listing flexibilities enabling widespread access to medicines [48].

### Compulsory licenses and public non-commercial use licenses

Examples of TRIPS flexibilities that WTO member states can use to pursue public health goals include granting compulsory licenses to third parties without patent holder consent and public non-commercial or government use of patents [41, 44]. These licenses allow local production or importation of generics [20]. Importantly, TRIPS does not require prior negotiations for a voluntary license in emergency or urgent situations [41]. In January 2017, WTO amended TRIPS with article 31(bis) to provide a special compulsory license to supply medicines to countries without sufficient production capacity [49]. Article 31(bis) allows Regional Economic Communities, consisting mostly of LDCs (all African), to bundle demand to supply the entire region, regardless of whether a developing country or an LDC will benefit.

Compulsory licensing (particularly non-commercial use licenses) facilitated procurement of generic ART for HIV when voluntary licenses were unavailable [50]. However, middle- and high-income countries that use compulsory licensing for DAAs are likely to experience significant opposition from manufacturers defending their monopoly and from countries where multinational pharmaceutical companies operate and manufacture [51]. To date, only Malaysia has issued a compulsory license for a DAA (late 2017) [44].

### Voluntary licenses

Pharmaceutical companies can establish agreements with generic manufacturers allowing the manufacture and sale of lower-priced generics before patent expiry [44, 52]. In 2010, the Medicines Patent Pool (MPP) was established to negotiate voluntary licenses with patent holders to supply ART in low- and middle-income countries [53]. MPP licenses will save the global health community an estimated US$ 2.3 billion for HIV treatment over 2010–2028 [53].

The MPP’s remit recently expanded to include DAAs, listing daclatasvir (GSK) in 2016. Prior to this expansion, Gilead established voluntary licensing agreements with 11 Indian companies, enabling them to manufacture and market bioequivalent DAAs in 101 low-income countries. Generic manufacturers with a voluntary license set their own price and pay royalties to Gilead [54]. These agreements excluded Brazil, China, Morocco and Thailand, middle-income countries with high hepatitis C burdens [55, 56]. However, countries can issue a compulsory license and produce or purchase generic medications, including from sub-licensees [51]. Interestingly, when Malaysia issued a compulsory license for sofosbuvir, Gilead responded by including Malaysia, Thailand, Ukraine and Belarus in their license agreement territory, showing the direct and indirect power of compulsory licensing [57].

Gilead Sciences has provided voluntary licenses for both ARTs and DAAs. Bristol-Myers Squibb has licensed daclatasvir to MPP for sale in 112 countries [58].

### Patent opposition

Pharmaceutical companies can be prevented from securing a monopoly using patent opposition [59]. In 2015, Médecins du Monde and civil society organisations from 17 countries challenged Gilead’s sofosbuvir patent, leading to amendment of the patent claims [60] and availability of generic versions of sofosbuvir in Europe in 2024 rather than 2028 [61]. Civil society organisations launched similar oppositions in Argentina, Brazil, China, Colombia, India, Ukraine and the US [61]. In 2012, Médecins Sans Frontières established a patent opposition database to disseminate information on claims and oppositions [62], an important tool for transparency and collaboration between public health groups and legal and technical experts on pharmaceutical patent challenges.

## Joint procurement

Governments can obtain lower pharmaceutical prices by allowing a subset of payers to negotiate prices as one entity [44, 63]. Benefits of joint procurement include allowing authorities to negotiate volume-based discounts, reap administrative savings and pool skillsets [44, 64]. Joint procurement can occur through collaborative agreements between contracting authorities or through permanent joint procurement organisations [64]. In 2015, Mercosur (a trade block involving Argentina, Brazil, Paraguay, Uruguay and Venezuela) and associated countries (Chile, Colombia, Peru and Ecuador) completed the first joint procurement of DAAs with support from the Pan American Health Organization Strategic Fund [65], securing the lowest price in the region (US$ 2292 per treatment) [66].

In 2017, the Centre for Disease Analysis Foundation established the Global Procurement Fund (GPRO) to support expanded access to affordable and quality treatments in low- and middle-income countries [67]. GPRO uses pooled purchasing to negotiate prices for large volumes of DAAs, with 104 countries currently accessing sofosbuvir through this mechanism [68]. Drugs supplied by GPRO must meet quality standards from WHO, a stringent regulatory authority or an independent expert review panel [67]. In 2008, the European Commission released a fact sheet to aid organisations to establish joint procurement [64]; however, this is yet to be employed for DAAs. Effective procurement of DAAs requires reliable surveillance and modelling data to predict the number of patients who need treatment [44].

## Personal importation schemes

In various countries (e.g. Australia [69], Italy [70] and Switzerland [71]), patients are legally entitled to import 1–3 months of personal medication. Patients living in countries without subsidised access to treatment can purchase generic DAAs through online buyers’ clubs [52]. The Australian-based FixHepC Buyers Club allows patients with a prescription to import a 12-week course of generic sofosbuvir and daclatasvir for US$ 1000 [72]. Cure rates of generic DAAs imported into Australia are similar to branded treatments [73]. However, in some countries, physicians cannot prescribe unlicensed medications [56]. Indeed, personal importation is not a long-term solution, with treatment uptake being somewhat ad hoc rather than part of a cohesive public health response.

## Case study 1: Australia

In Australia, the Therapeutic Goods Administration (TGA) regulates the pharmaceutical sector [74] and the Pharmaceutical Benefits Scheme (PBS) subsidises listed medications for Australian residents [75]. General and concession patients make co-payments of AU$ 39.50 and AU$ 6.40 per script, respectively [76]. If a TGA-approved drug is not listed on the PBS, Australians can still access these medications, although generally at full price. Pharmaceutical companies can apply for PBS listing through the Pharmaceutical Benefits Advisory Committee (PBAC) [75]. If the PBAC recommends the drug, the Department of Health negotiates a subsidised price with the pharmaceutical company [75].

In 2014, the TGA approved sofosbuvir for the wholesale cost of AU$ 110,000 per course. After long price negotiations between the government and pharmaceutical companies, PBAC recommended DAAs for PBS listing on March 1, 2016. The Australian government budgeted approximately AU$ 1.2 billion over 5 years to treat hepatitis C [56], following a volume-based, risk-sharing deal with pharmaceutical companies [56]. Treatment costs are capped at approximately AU$ 250 million annually, regardless of the number of people treated [77], yet the ‘base price’ the government pays per treatment is thought to be approximately AU$ 12,000–15,000. However, if annual treatment costs exceed the annual cap, these are covered by the pharmaceutical companies; therefore, the more patients treated, the lower the cost per course, which incentivises treatment scale-up. Between March 2016 and June 2017, approximately 43,360 patients (19% of all Australian patients with hepatitis C) were treated [78] at an estimated cost of less than US$ 8000 per course, one of the lowest prices per patient in high-income countries.

Importantly, prescribing guidelines allow treatment by specialists, general practitioners and nurse practitioners, facilitating uptake of treatment outside hospital settings [79]. Further, fibrosis and substance use do not affect eligibility [79]. Based on PBS co-payment schemes, general patients pay out-of-pocket AU$ 120 and concession patients pay AU$ 20 per treatment course [76], making the DAA price for individual patients one of the lowest globally.

## Case study 2: Portugal

Initially, in Portugal, DAAs cost approximately € 40,000 per patient [12] and treatment was restricted to patients with severe cirrhosis who required a liver transplant, which led to community advocacy in the media and parliament [80] designed to shift discussions about hepatitis C from prevention to cure [80]. In February 2015, the Portuguese government announced a risk-sharing and volume-based agreement with Gilead, enabling universal access to treatment for all patients, regardless of fibrosis stage [80, 81]. Gilead would initially only receive payment per patient (less than € 7000) if treatment led to a cure. A national registry was established to monitor cure rates 12 weeks post-treatment [81]. As of July 2017, 17,591 patients had been authorised treatment, 11,972 patients had initiated treatment and 6639 (96.5%) patients were clinically cured [82]. The programme has averted 3477 premature liver deaths, 339 liver transplants and 5417 cases of cirrhosis [83], and saved the government over € 271 million on treatment of hepatitis C complications [83]. A national plan to improve care is currently under development [84].

## Case study 3: Egypt

Egypt has the highest hepatitis C prevalence worldwide [85], mostly attributable to transmission through injections to treat schistosomiasis between the 1950s and 1980s [85]. In 2008, an estimated 9.8% of Egypt’s population aged 15–59 years was hepatitis C positive; prevalence was highest among those aged 50–59 [86]. In 2015, the estimated prevalence among those aged 15–59 years declined to 7.0% [87], predominantly due to ageing of the population with hepatitis C [88, 89].

In 2006, the National Committee for Control of Viral Hepatitis was established to measure hepatitis C burden and prepare a national treatment programme [90] and, in 2014, a national plan for prevention, control and treatment of viral hepatitis was released. The National Committee for Control of Viral Hepatitis and the Health Insurance Organization set up 189 government treatment facilities and created a national online database to register patients [91]. Importantly, the Egyptian government rejected patent applications for sofosbuvir, enabling local production and supply of generics [92]. In 2014, Egypt had a limited supply of branded sofosbuvir, which cost US$ 900 for a 12-week treatment course – still expensive relative to Egypt’s financial resources [91]. Treatment was restricted to patients with advanced fibrosis, hepatitis B or HIV co-infection, and post-liver transplants, leading to long waiting lists [91]. Subsequently, negotiations and generic production reduced prices significantly and DAAs became available to all patients [91]. Between October 2014 and December 2017, 1.4 million patients commenced treatment, with cure rates above 90% [91]. Because there are no compound patents on sofosbuvir and daclatasvir in Egypt [93], treatment is now available for US$ 84 per treatment course from generic manufacturers [21]. In 2016, the Ministry of Health established the Viral Hepatitis Control Administration, aiming to reduce new infections, screen 10 million people, eliminate waiting lists and enhance their sentinel surveillance systems [94]. Despite high hepatitis C prevalence, Egypt is on track to achieve WHO elimination targets [1].

## Ways forward and future challenges

To achieve the WHO 2030 elimination targets, DAA prices, including those governments or insurers pay per course and for individual patients, must generally fall to ensure universal access [95]. Governments need to negotiate drug prices and be prepared to use the TRIPS flexibilities to ensure satisfactory results. In countries such as Denmark and the US, restrictions on negotiating drug prices need to be removed. Up-to-date public information about DAA availability and pricing is also important [11]; such transparency shows governments that pharmaceutical companies are willing to negotiate a price that enables elimination. Since 1986, Management Sciences for Health and WHO have published the International Drug Price Indicator Guide, which lists drug prices from non-profit and procurement agencies – a valuable tool for tackling drug pricing [96].

## Public health approach in negotiations

Drug price negotiations appear to be cyclical. Upon marketing approval, pharmaceutical companies often set high prices [97]; governments then enter negotiations with manufacturers, often with a fixed budget [40]. Reliable data on drug development and manufacturing costs would assist governments to negotiate reasonable prices [98] that take into account companies’ justifiable need for profits. When drug development and manufacturing costs have been recovered, governments may have an easier time negotiating prices, enabling them (as in the case of DAAs) to quickly adopt a public health approach rather than an individual patient approach.

Pharmaceutical companies have shown preparedness to negotiate reasonable prices for DAAs, particularly when countries have comprehensive public health-based viral hepatitis elimination plans that facilitate treatment access for a large proportion of the affected population. Gilead has demonstrated its willingness to provide voluntary licenses that enable certain countries to access DAAs at a reduced price, as has GSK through the MPP.

Comprehensive viral hepatitis plans have centred on treatment as prevention and hepatitis C elimination rather than treating individual patients. These plans must be supported by reliable estimates of the number of people needing treatment so that governments can set elimination targets and monitor treatment outcomes. However, there are challenges in establishing hepatitis C surveillance systems (e.g. cost, establishing case definitions, laboratory infrastructure, training staff) [99], yet governments can learn from countries with surveillance systems in place (e.g. Australia, Egypt, Portugal).

## Conclusion

History records very few opportunities to eliminate a chronic infection. In the DAA era, eliminating hepatitis C as a public health threat is possible, yet it can only be achieved with affordable access to DAAs worldwide. Case studies of Australia, Portugal and Egypt demonstrate that comprehensive public health-based viral hepatitis plans facilitate negotiations with pharmaceutical companies. Shifting from individual-focused hepatitis C treatment to elimination requires strong political will and advocacy. If price negotiations with pharmaceutical companies do not produce reasonable prices for DAAs, governments can utilise flexibilities in patent law to ensure access to low-priced generic sources.

## Abbreviations

ART: antiretroviral therapy
DAAs: direct-acting antivirals
FDA: Food and Drug Administration
GPRO: Global Procurement Fund
LDCs: least developed countries
MPP: Medicines Patent Pool
PBAC: Pharmaceutical Benefits Advisory Committee
PBS: Pharmaceutical Benefits Scheme
PWID: people who inject drugs
R&D: research and development
TGA: Therapeutic Goods Administration
TRIPS: Trade-Related Aspects of Intellectual Property Rights
US: United States
WHO: World Health Organization
WTO: World Trade Organization
